# Evaluating Covid-19 publications for sex and gender-specific health content: A bibliometric analysis

**DOI:** 10.1371/journal.pone.0316812

**Published:** 2025-02-19

**Authors:** Abigail Oyasu, Aysha Salter-Volz, Chen Yeh, Lutfiyya N. Muhammad, Reshma Jagsi, Nicole C. Woitowich

**Affiliations:** 1 Department of Medical Social Sciences, Feinberg School of Medicine, Northwestern University, Chicago, Illinois, United States of America; 2 Department of Preventive Medicine, Feinberg School of Medicine, Northwestern University, Chicago, Illinois, United States of America; 3 Department of Radiation Oncology, Emory University, Atlanta, Georgia, United States of America; Max Planck Institute for Solid State Research, GERMANY

## Abstract

**Background:**

Sex and gender are key variables which inform human health and disease. It remained unclear how sex and gender were considered, evaluated, reported, or analyzed within Covid-19 research. This article evaluates the proportion of Covid-19-related articles which highlighted sex- or gender-specific health content and examines associations with author gender.

**Materials and methods:**

Article records for 134,008 publications indexed in the LitCovid database were extracted on June 1^st^, 2021. Metadata such as publication year, author names, and country of institutional affiliation were obtained from Elsevier’s SCOPUS database by matching PubMed Identifiers (PMIDs). Only articles with matching SCOPUS records were included in the study, resulting in a final sample of 94,488 articles. First and last author gender was assigned to a subset of 71,597 articles. Article title, abstracts, and keywords were screened for sex or gender-specific health content using a text-based search strategy. Descriptive statistics and regression analyses were used to study associations between author gender and the presence or absence of sex or gender-related health content.

**Results:**

Only 4% of Covid-19-related articles highlighted sex or gender-related health content. Papers with women first authors were more likely to highlight sex or gender-related health content compared to papers with men as first authors (4.15% n = 1,339 vs 3.68%, *n* = 1,997) [*X*^2^ (1, *n* = 86,468) = 12,01, *p* = 0.0005]. Papers with women first and last authors had an increased probability of addressing sex or gender-related health with an odds ratio of 1.16 (95% CI 1.04 – 1.29). While there was no association between author gender and journal impact, articles which highlighted sex or gender-related health content were published in journals with higher CiteScores [Mdn = 5.0, Q1-Q3 (3.5–8.2) vs. Mdn = 4.7, Q1-Q3 (2.8–8.0)].

**Conclusions:**

The paucity of publications to highlight sex or gender in the context of the Covid-19 pandemic is alarming. Research that focuses on the influence of sex and or gender is essential for advancing the scientific understanding of disease processes.

## Introduction

Our current understanding of health and disease is derived from decades of biomedical research, education, and training centered on the male sex [1]. This hinders our understanding of normal physiological processes as well as how disease mechanisms, manifestations, and treatment are affected by the complex relationship between sex and gender. Moreover, it perpetuates biases in the way biomedical research is conducted, analyzed, and reported [2–4]. Further downstream, a lack of consideration for sex and gender-based factors impacts patient outcomes and clinical care leading to poorer health outcomes for women and gender minorities in particular [5–11]. Sex and gender-inclusive research is therefore important both to improve the health of understudied groups and to optimize the rigor of science in ways likely to benefit all.

Despite numerous calls to action [12–17] and the implementation of policies to address sex and gender-inclusive research [18,19], bibliometric analyses have revealed inconsistencies in the way sex and gender are considered, reported, or analyzed within biomedical research [20–24]. Moreover, they suggest that women-led research teams are the driving force behind sex and gender-inclusive research practices [21,22,25]. This presents a practical challenge for advancing research focused on sex and gender-specific health (SGSH) as women are underrepresented within the biomedical workforce, particularly within leadership roles associated with research design and development [26,27]. Exploring how gender influences research practices and decision-making is a critical component of understanding how SGSH can improve.

The Covid-19 pandemic provided a unique context in which to examine SGSH across the biomedical research enterprise. Early on during the Covid-19 pandemic, sex and gender differences in disease prevalence, symptomology, and response to treatment emerged [28]. It is well-known that there are sex differences in immune response and susceptibility to viral infection [29,30]. There are also gender differences in exposure to pathogens, health behaviors, and access to health care which contribute to morbidity, mortality, and disease heterogeneity [31]. Yet, it remains unclear how SGSH was considered within Covid-19 research. Early bibliometric reports also suggested that women were underrepresented as authors of Covid-19 research, due to an increase in household responsibilities during the pandemic [32]. If women were unable to fully participate in the design, development, and dissemination of Covid-19 research, could this change how sex and gender were considered? Using a bibliometric approach, we sought to address these gaps. Although Covid-19 may no longer be the threat it once was, understanding how research proceeded during that pandemic has the potential to illuminate ongoing challenges and opportunities for intervention.

## Methods

### Overview

This bibliometric analysis was designed to assess if sex or gender-specific health content was a focus in Covid-19-related publications; to test associations between author gender and the presence or absence of sex or gender-specific health content; and to examine other factors associated with the presence or absence of sex or gender-specific health content.

### Ethics approval and consent to participate

This study did not involve human subjects and therefore did not require ethical approval or consent for participation.

### PubMed

**Fig 1:**
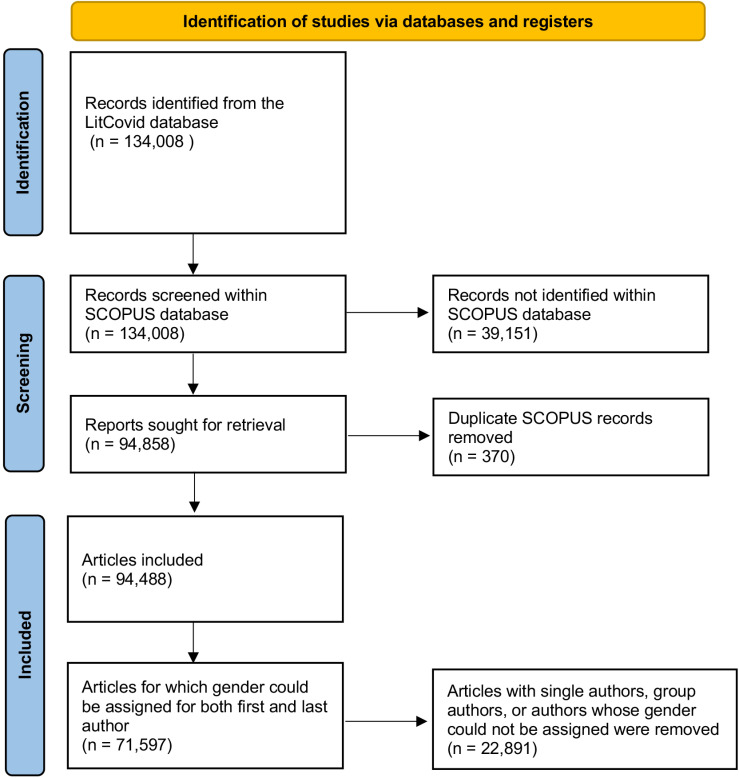
Preferred Reporting Items for Systematic Reviews and Meta-Analyses (PRISMA) flow-chart of publication record selection.

Data for this project were obtained from LitCovid, a curated database of Covid-19-related publications maintained by the National Center for Biotechnology Information (NCBI) [33,34]. Articles are sourced daily using PubMed’s E-Utilities tool and are screened and classified via a combination of machine learning and manual curation. All publication types, with the exception of preprints, are included within the LitCovid database. Article records (N = 134,008) were downloaded from LitCovid on June 1st, 2021 (Fig 1).

### Scopus

Each article record downloaded from the LitCovid database contained the Pubmed Identifier (PMID), article title, and journal title. Next, publication metadata was obtained from Elsevier’s SCOPUS database (https://www.scopus.com/) by matching PMIDs. This resulted in a total of 94,488 unique publication records containing the following information: publication year, first and last author names, country of institutional affiliation for the first and last author, and the total number of authors per record. In addition, each record contained the corresponding journal’s CiteScore, a citation impact metric developed by Elsevier [35].

### Gender assignment of authors

Author first (given) names and country of institutional affiliation were used to assign author gender using GenderAPI (https://gender-api.com/). GenderAPI was chosen based on its low rate of mis-classifications and non-classifications [36]. It assigns gender as either male, female, or unknown. Authors listed by their first initial were manually coded as unknown (n = 6,093 and n = 5,775 for first and last authors, respectively). Articles which contained multiple first or last authors were coded as group authors (n = 207 and n = 1,904, respectively). When first names were available, the number of non-classifications were low (n = 1,720 and n = 1,629 for first and last authors, respectively). Gender API provides a reliability score that quantifies the likelihood that the name is male or female. We provide a description of sample by reliability score by first and last author (S1 and S2 Tables). When gender could be assigned for both the first and last authors (n = 71,597), these articles were coded by author-dyad as: Man-Man (MM), Man-Woman (MW), Woman-Man (WM), or Woman-Woman (WW).

Because the terms male and female typically refer to biological sex, the terms man/men or woman/women are utilized here. However, it is important to note that gender is a social construct that is non-binary and non-static. Authors whose gender identity is non-binary or whose gender identity has changed since the time of publication are at risk of being misgendered. This remain a major limitation of this type of name-to-gender methodology.

### Analysis of sex and gender specific health content

In addition, each article was screened for sex and gender specific health (SGSH) content by using the following search strategy: “‘TITLE-ABS-KEY(“sex difference* OR TITLE-ABS-KEY(“sex based”) OR TITLE-ABS-KEY(“sex factor*”) OR TITLE-ABS-KEY(“sex distribution”) OR TITLE-ABS-KEY(“sex characteristic*”) OR TITLE-ABS-KEY(“sex dimorphism”) OR TITLE-ABS-KEY(“gender difference*”) OR TITLE-ABS-KEY(“gender based”). This strategy was based on the Texas Tech University Health Sciences Center (TTUHSC) SGSH PubMed Search Tool developed by Song and colleagues [37] to identify literature which explores the influences of sex and gender on health. Unlike the TTUHSC SGSH PubMed Search Tool, this strategy did not require the inclusion of the terms sex or gender in the article title, potentially reducing its sensitivity but broadening its scope.

To evaluate the search strategy for false negatives (articles in which SGSH content was included albeit not highlighted in the title, abstract or keywords), a stratified sample of 60 articles (15 from each of the author-dyad categories) which did not highlight SGSH content were selected for manual review (S3 Table). Of those, 44 did not include the terms sex or gender anywhere in the article. Of the 16 articles that included the terms sex or gender, only 3 contained SGSH content. Two articles did not include terms sex or gender within the article but were found to contain SGSH content.

A similar strategy was used to identify false positives (Supplemental S3 Table). A stratified sample of 60 articles (15 from each of the author-dyad categories) which included SGSH content were manually reviewed. Of the 60 articles, 3 did not include the terms sex or gender anywhere in the article, however one contained SGSH content describing the differences in Covid-19 morbidity and mortality using the terms: male, female, man/men, or woman/women. Two articles contained the terms sex or gender but upon review, did not contain SGSH content: One of the articles described a study design as gender-balanced, while another article described a multivariable logistic regression model considering gender but provided no additional content or context.

### Statistical analyses

Data were analyzed using SAS software version 9.4 and GraphPad Prism version 10.0.2. Descriptive statistics were calculated for all variables of interest. Categorical variables were summarized with frequencies and percentages, and continuous variables were summarized with medians (Mdn) and interquartile ranges. Chi-Square tests were used to compare author gender and articles highlighting SGSH content. The Wilcoxon test for continuous variables of interest was used to test the association between highlighting SGSH content and CiteScore. *P*-values < 0.05 were considered significant.

### Regression analysis

In the SGSH Content model, the dependent variable is a binary variable that indicates the presence or absence of SGSH content within an article’s title, abstract, keywords (hereinafter described as “highlighting SGSH content”). Logistic regression models were fitted for author gender using the author dyad categories of Man-Man, Man-Woman, Woman-Man, and Woman-Woman with Man-Man as the reference. Odds ratios (OR) and 95% confidence intervals were used to summarize the logistic regression model findings.

## Results

Of the 94,488 COVID-19-related articles in this sample, only 3.81% (*n* = 3,602) highlighted SGSH content (Table 1). A significantly higher proportion of articles highlighted SGSH content when the first author was a woman (4.15% n = 1,339) compared to a man (3.68%, *n* = 1,997) [*X*^2^ (1, *n* = 86,468) = 12,01, *p* = 0.0005; Table 2). However, there were no significant differences in the proportion of articles highlighting SGSH content by last author gender (woman, 4.25% n = 927 vs. man 4.04%, n = 2,094).

**Table 1:** Characteristics of Covid-19-related publications.

	N	%
**First Author Gender**	94,488	100
Man	54.222	57
Woman	32,246	34
Unknown	7,813	8
Group	207	1
**Last Author Gender**	94,488	100
Man	51,828	55
Woman	21,792	23
Unknown or Not Applicable	18,694	20
Group	1,904	2
**Author Dyads**	71,597	100
Man first/ Man last	33,479	47
Man first/ Woman last	11,067	15
Woman first/ Man last	16,913	24
Woman first/ Woman last	10,138	14
**First Author Country of Institutional Affiliation**	94,488	100
Asia	24,008	25
Africa	2,000	2
Europe	30,572	32
North America	25,467	27
Oceania	2,219	2
South America	3,553	4
Missing	6,669	7
**Included Sex or Gender Specific Health Content**	94,488	100
No	90,886	96
Yes	3,602	4
**Other Characteristics**	**N**	**Mdn (Q1-Q3)**
Number of Authors	94,448	5.0 (3.0-8.0)
CiteScore	94,338	4.7 (2.9-8.0)

**Table 2:** Comparison of Covid-19 publications by highlighting of Sex and Gender-Specific Health (SGSH) content by author gender.

		No highlighted SGSH content		Highlighted SGSH content		
	Total	N	%	N	%	p-value
**First Author Gender**	86,468	83,132	96.14	3,336	3.86	0.0005
Man	54,222	52225	96.32	1997	3.68	
Woman	32246	30907	95.85	1339	4.15	
**Last Author Gender**	73,620	70599	95.90	3021	4.10	0.1824
Man	51,828	49734	95.96	2094	4.04	
Woman	21,792	20865	95.75	927	4.25	
**Author Dyads**	71,597	68654	95.89	2943	4.11	0.0028
Man first/ Man last	33,479	32161	96.06	1318	3.94	
Man first/ Woman last	11,067	10632	96.07	435	3.93	
Woman first/ Man last	16,913	16182	95.68	731	4.32	
Woman first/ Woman last	10,138	9679	95.47	459	4.53	

**Fig 2:**
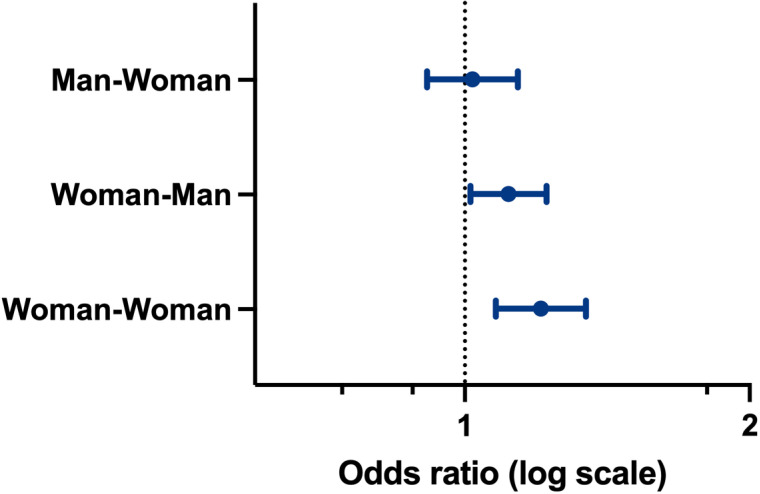
Odds ratio of articles highlighting sex and gender-specific health content from the logistic regression analysis. The reference variable for gender of first and last authors is man-man, Error bars are 95% CI.

From the total sample, the gender of first and last author dyads was available for 71,597 articles (Tables 1 and 2). Articles were excluded from this sample if they were authored by a single individual, authored by a group in either the first or last author position, or if the gender of either the first or last author could not be determined. There was a significant difference in the proportion of articles which highlighted SGSH content by the gender of the author dyad [*X*^2^ (1, *n* = 71,597) = 8.91, *p* = 0.0028.] Man-first/man-last dyads accounted for 47% (*n* = 33,479) of articles, with 3.94% (*n* = 1,318) of these highlighting SGSH content. Man-first/woman-last dyads represented 15% (*n* = 11,067) of articles, with 3.93% (*n* = 435) of these highlighting SGSH content. Woman-first/man-last dyads accounted for 24% (*n* = 16,913) of articles, with 4.32% (*n* = 731) of these highlighting SGSH content. Lastly, Woman-first/woman-last dyads comprised 14% (*n* = 10,138) of articles, with 4.53% (n = 459) highlighting SGSH content. In an unadjusted model, having women first and last authors was positively associated with the highlighting of SGSH content, with an OR of 1.16 (95% CI 1.04 − 1.29; Fig 2). When considering the total sample, irrespective of author gender, articles that highlighted SGSH content were published in journals with higher CiteScores compared to those that did not [Mdn = 5.0, Q1 − Q3 (3.5 − 8.2) vs. Mdn = 4.7, Q1 − Q3 (2.8 − 8.0); p < 0.001; Table 3].

**Table 3:** Comparison of Covid-19 publications by Highlighting of Sex and Gender-Specific Health (SGSH) content and citescore.

	No highlighted SGSH content (n = 90,739)	Highlighted SGSH content (n = 3,599)	p-value
CiteScore OR (95% CI)	4.70 (2.8 – 8.0)	5.0 (3.5 – 8.2)	< 0.0001

## Discussion

Even as the acute phase of the Covid-19 pandemic recedes into history, there remains a clear need to advance our understanding of how sex and gender influences human health and disease [38]. However, the implementation of education, research, and reporting practices to do so has been lackluster across many disease conditions, and Covid-19 was no exception, despite early evidence that the condition affected men and women differently and involved inflammatory and immune responses that varried by sex and gender. This bibliometric analysis reveals that only 4% of Covid-19-related articles highlighted the impact of sex or gender. Interestingly, our results are analogous to a 2021 study by Brady and colleagues which found that only 4% of Covid-19 clinical trials planned to analyze data by sex and gender [39]. These data, when viewed through the lens of a global public health crisis with clear gendered impact, underscore the lack of attention to sex and gender-specific health.

Our data reveal that articles with women first authors and articles with both women first and last authors are more likely to highlight SGSH content. This is consistent with findings by several other groups who have explored the interaction between author gender and sex/gender reporting and analyses [21,22,25,40]. Recently, Carrillo and colleagues examined the association between author gender and sex reporting and analyses in a sample of Covid-19-related articles published in Spanish medical journals. They found that women first authors were 1.5 times more likely to disaggregate data by sex [40]. When evaluating the global research landscape, Merriman and colleagues reported that articles with women first and last authors were almost 2.5 times more likely to discuss or address sex and gender [25]. Here we report that articles with women first and last authors were 1.2 times more likely to highlight sex or gender-related content. The difference in effect sizes may be based on our larger dataset (71,597 vs 557 and 542 articles) [25,40]. Previous work by Sugimoto and colleagues suggests that this may be the case: they examined over 1.1 million articles and similarly found that women-led articles were 1.2 times more likely to incorporate sex-related reporting [22]. Alternatively, the difference in effect sizes may be due to the methods used to identify sex- or gender-related content. Our focus was on those articles in which SGSH content was sufficiently robust to warrant highlighting in the title, abstract, or keywords. Both Carrillo et al., and Merriam et al., utilized a manual coding strategy to identify SGSH content, which is likely more accurate and sensitive than the *in silico* methods that we used [25,40]. We posit that there is a trade-off between the accuracy of identifying SGSH content and the total number of articles in the sample, resulting in the differences in effect size. Regardless, this work contributes to a growing body of literature which suggests that women are more likely to focus on sex and gender within the context of their research.

We also found that women constituted a minority of the authors of Covid-19-related articles, occupying 34% and 23% of first and last author positions, respectively. Prior research revealed that women’s representation among authors of Covid-19 research was lower than of other research published previously in those same journals, suggesting that the disruptions of the pandemic may have had a disproportionate impact on women researchers [41]. Several other studies have examined author-level data of Covid-19-related publications [40,42–44]. They found that the proportion of women first authors ranged from 33-38%, while the proportion of women last authors ranged from 28–34%. The proportion of women last authors in our study was notably lower at 23%. This may be due, in part, to differences in sample sizes between our work and the previously published studies (94,488 vs. 914, 15,843 and 42,898) [40,43,44] as well as the types of articles included within the search strategy. Specialty-specific studies in cardiology [45], pulmonary and critical care [46] have also revealed gender disparities in authorship of Covid-19-related articles and manuscript acceptance rates, respectively. Prior to the pandemic, women in biomedicine were more likely to take on additional household or care responsibilities [47,48]. During the pandemic, these gender disparities were amplified to an unprecedented degree [49]. Women were more likely to experience professional setbacks, such as a loss of research productivity [50]. This particularly impacted women with young children, many of whom are early-career scientists who rely on publications for their professional advancement [51]. The current study is especially important in highlighting how the underrepresentation of women as authors of Covid-19 research can have meaningful impact on the nature of the science that emerges. This finding underscores the importance of addressing the disproportionate impact of family caregiving on certain subgroups in the STEMM workforce whose contributions are essential, as recently addressed in a landmark report from the National Academies of Sciences, Engineering, and Medicine [52]. Moreover, our findings suggest that given the fact that that SGSH is fundamental to understanding mechanisms of disease and informing approaches to promote the health of all members of our society, further efforts are needed to ensure that in the future this work is led by all, and not disproportionately by women.

Interestingly, we found that articles highlighting SGSH content were more likely to be published in high impact journals. This was an unexpected finding, in contrast to the work by Sugimoto and colleagues, which found that articles containing sex-related reporting were more likely to be published in low impact journals [22]. Presently, only one-third of top tier medical journals have required sex and/or gender reporting standards [53]. Because the publications in our dataset only pertained to Covid-19, sex and gender-specific content may have addressed public health imperatives, warranting publication in a high impact journal. While our data is encouraging, the lack of editorial policies regarding sex/gender reporting and analyses, or the lack of their enforcement, remains a barrier to advancing SGSH research.

It is important to note that this study is not without limitations. First, the bibliometric dataset obtained from LitCovid contained all types of articles related to Covid-19 including research articles, case reports, editorials, opinion pieces, and review articles. This reduces the proportion of articles directly involving human or animal subjects in the total sample, skewing the likelihood that an article contained SGSH content of the sort that could be highlighted. Assigning gender to authors, based on name and country-level data is a common limitation of bibliometric analyses. The *in silico* programs used to assign gender based on first names are not ideal, as they reduce gender to a binary, and do not capture the representation of authors who identify as transgender or gender non-binary. In addition, Asian names have higher rates of misclassification compared to names of other origins, as do unique or “gender-neutral” names which may have been coded as “unknown” and excluded from analyses [36].

The search strategy used to determine the presence or absence of highlighted SGSH content is a known limitation. However, we conducted a manual review of articles to ascertain the rate of false positives or negatives. Discrepancies were found regarding articles that included the words sex or gender but were not flagged as containing SGSH content and vice versa. Articles may have included SGSH content in the title or abstract but did not include explicitly state terms such as “sex distribution” or “gender difference”, for example. This suggests the SGSH PubMed Search Tool is sufficient at recognizing SGSH terms, but insufficient at quantifying the extent of sex and gender analysis. As text- and data-mining tools become more robust, the identification of articles based on nuanced content will likely improve these search strategies. Nevertheless, our results appear to have captured with reasonable accuracy those articles in which SGSH was a highlighted focus. Lastly, the relationship between author gender and the highlighting of SGSH content is correlative and causality cannot be determined based on the observational nature of this study.

The data presented here underscores two findings of great importance to the biomedical research enterprise: First, the lack of focused attention to sex and gender in biomedical research is detrimental to the advancement of health and well-being on a global scale. Second, our findings indicate that women are disproportionately responsible for considering and addressing sex and/or gender within their research. Research pertaining to SGSH is relevant and important to the health and well-being of all people. Members of the biomedical research enterprise, including funders, publishers, and policymakers, need to work collectively to address sex and gender biases that persist within our community across all levels. This is essential to advance our understanding of human health and disease and to foster an equitable, inclusive biomedical workforce.

## Supporting information

S1 Table A) Comparison of gender API reliability score by first author gender. (PDF)

S2 Table B) Comparison of gender API reliability score by last author gender. (PDF)

S3 Table C) Manual validation of Sex and Gender Specific Health (SGSH) search strategy. (PDF)
