# Supplementary material for: Evaluating Covid-19 publications for sex and gender-specific health content: A bibliometric analysis
Source: PLoS One. 2025 Feb 19;20(2):e0316812. doi: 10.1371/journal.pone.0316812 (PMC11838872; doi:10.1371/journal.pone.0316812)
Supplement: S1 Table — (PDF) [file pone.0316812.s001.pdf]

Supporting Information

Table A. Comparison of Gender API Reliability Score by First Author Gender

Gender Reliability Score	Male N	Male %	Female N	Female %	Unknown* N	Unknown* %	Total N	Total %
≥ 0.90	45549	*84.0*	26763	*83.0*	0	*0.0*	72312	*82.0*
0.80 - 0.89	2527	*4.7*	1567	*4.9*	0	*0.0*	4094	*4.6*
0.70 - 0.79	2992	*5.5*	1726	*5.4*	0	*0.0*	4718	*5.3*
0.60 - 0.69	2028	*3.7*	1139	*3.5*	0	*0.0*	3167	*3.6*
0.50 - 0.59	1126	*2.1*	1051	*3.3*	455	*26.0*	2632	*3.0*
Not Scored	0	*0.0*	0	*0.0*	1265	*74.0*	1265	*1.4*

*Excludes articles with authors listed by their first initial only.
