# Supplementary material for: Evaluating Covid-19 publications for sex and gender-specific health content: A bibliometric analysis
Source: PLoS One. 2025 Feb 19;20(2):e0316812. doi: 10.1371/journal.pone.0316812 (PMC11838872; doi:10.1371/journal.pone.0316812)
Supplement: S2 Table — (PDF) [file pone.0316812.s002.pdf]

Table B. Comparison of Gender API Reliability Score by Last Author Gender

| Gender Reliability Score | Male | | Female | | Unknown* | | Total | |
|---|---|---|---|---|---|---|---|---|
| | N | % | N | % | N | % | N | % |
| ≥ 0.90 | 44288 | *85.5* | 18024 | *82.7* | 0 | *0.0* | 62312 | *82.8* |
| 0.80 - 0.89 | 2024 | *3.9* | 943 | *4.3* | 0 | *0.0* | 2967 | *3.9* |
| 0.70 - 0.79 | 2920 | *5.6* | 1190 | *5.5* | 0 | *0.0* | 4110 | *5.5* |
| 0.60 - 0.69 | 1765 | *3.4* | 777 | *3.6* | 0 | *0.0* | 2542 | *3.4* |
| 0.50 - 0.59 | 831 | *1.6* | 858 | *3.9* | 392 | *24.1* | 2081 | *2.8* |
| Not Scored | 0 | *0.0* | 0 | *0.0* | 1237 | *75.9* | 1237 | *1.6* |

*Excludes articles with authors listed by their first initial only.
