# Supplementary material for: Evaluating Covid-19 publications for sex and gender-specific health content: A bibliometric analysis
Source: PLoS One. 2025 Feb 19;20(2):e0316812. doi: 10.1371/journal.pone.0316812 (PMC11838872; doi:10.1371/journal.pone.0316812)
Supplement: S3 Table — (PDF) [file pone.0316812.s003.pdf]

Table C. Manual validation of Sex and Gender Specific Health (SGSH) Search Strategy

	**Appearance of terms "sex" or "gender"**	**Inclusion of SGSH content**
**Absence of SGSH content (false negatives) (n=60)**	27% (n=16)	10% (n=6)
**Presence of SGSH content (false positives) (n=60)**	95% (n=57)	93% (n=56)
